# Connectivity in the Yeast Cell Cycle Transcription Network: Inferences from Neural Networks

**DOI:** 10.1371/journal.pcbi.0020169

**Published:** 2006-12-22

**Authors:** Christopher E Hart, Eric Mjolsness, Barbara J Wold

**Affiliations:** 1 Division of Biology, California Institute of Technology, Pasadena, California, United States of America; 2 Institute for Genomics and Bioinformatics, School of Information and Computer Science, University of California Irvine, Irvine, California, United States of America; 3 Biological Network Modeling Center, Beckman Institute, California Institute of Technology, Pasadena, California, United States of America; Technical University of Denmark, Denmark

## Abstract

A current challenge is to develop computational approaches to infer gene network regulatory relationships based on multiple types of large-scale functional genomic data. We find that single-layer feed-forward artificial neural network (ANN) models can effectively discover gene network structure by integrating global in vivo protein:DNA interaction data (ChIP/Array) with genome-wide microarray RNA data. We test this on the yeast cell cycle transcription network, which is composed of several hundred genes with phase-specific RNA outputs. These ANNs were robust to noise in data and to a variety of perturbations. They reliably identified and ranked 10 of 12 known major cell cycle factors at the top of a set of 204, based on a sum-of-squared weights metric. Comparative analysis of motif occurrences among multiple yeast species independently confirmed relationships inferred from ANN weights analysis. ANN models can capitalize on properties of biological gene networks that other kinds of models do not. ANNs naturally take advantage of patterns of absence, as well as presence, of factor binding associated with specific expression output; they are easily subjected to in silico “mutation” to uncover biological redundancies; and they can use the full range of factor binding values. A prominent feature of cell cycle ANNs suggested an analogous property might exist in the biological network. This postulated that “network-local discrimination” occurs when regulatory connections (here between MBF and target genes) are explicitly disfavored in one network module (G2), relative to others and to the class of genes outside the mitotic network. If correct, this predicts that MBF motifs will be significantly depleted from the discriminated class and that the discrimination will persist through evolution. Analysis of distantly related Schizosaccharomyces pombe confirmed this, suggesting that network-local discrimination is real and complements well-known enrichment of MBF sites in G1 class genes.

## Synopsis

A current challenge is to develop computational approaches to infer gene network regulatory relationships by integrating multiple types of large-scale functional genomic data. This paper shows that simple artificial neural networks (ANNs) employed in a new way do this very well. The ANN models are well-suited to capitalize on natural properties of gene networks in ways that many previous methods do not. Resulting gene network connections inferred between transcription factors and RNA output patterns are robust to noise in large-scale input datasets and to differences in RNA clustering class inputs. This was shown by using the yeast cell cycle gene network as a test case. The cycle has multiple classes of oscillatory RNAs, and Hart, Mjolsness, and Wold show that the ANNs identify key connections that associate genes from each cell cycle phase group with known and candidate regulators. Comparative analysis of network connectivity across multiple genomes showed strong conservation of basic factor-to-output relationships, although at the greatest evolutionary distances the specific target genes have mainly changed identity.

## Introduction

Hundreds of yeast RNAs are expressed in a cell cycle–dependent, oscillating manner. In both budding yeast and fission yeast, these RNAs cluster into four or five groups, each corresponding roughly to a phase of the cycle [1–9]. Large sets of phase-specific RNAs are also seen in animal and plant cells [10–12], arguing that an extensive cycling transcription network is a fundamental property of Eukaryotes. The complete composition and connectivity of the cell cycle transcription network is not yet known for any eukaryote, and many components may vary over long evolutionary distances [3–5,13], but some specific regulators (e.g., MBF of yeast and the related E2Fs of plants and animals) are paneukaryotic, as are some of their direct target genes (DNA polymerase, ribonucleotide reductase). Coupled with experimental accessibility, this conservation of core components and connections make the yeast mitotic cycle an especially good test case for studies of network structure, function, and evolution.

To expose the underlying logic of this transcription network, a starting point is to decompose the cell cycle into its component phases (i.e., G1, S, G2, M) and link the pertinent regulatory factors with their immediate regulatory output patterns, here in the form of phasic RNA expression. One way to do this is to integrate multiple genome-wide data types that impinge on connection inference, including factor:DNA interaction data from chromatin IP (ChIP) studies, RNA expression patterns, and comparative genomic analysis. This is appealing partly because these assays are genome-comprehensive and hypothesis-independent, so they can, in principle, reveal regulatory relationships not detected by classical genetics. However, the scale and complexity of these datasets require new methods to discover and rank candidate connections, while also accommodating considerable experimental and biological noise (e.g., [14–19]). Microarray RNA expression studies in budding yeast have identified 230 to 1,100 cycling genes, the upper number encompassing nearly a fifth of all yeast genes [1,2,8,20]. Specifics of experimental design and methods of analysis contribute to the wide range in the number of genes designated as cycling, but there is agreement on a core set of nearly 200. Yeast molecular genetic studies have established that transcriptional regulation is critical for controlling phase-specific RNA expression for some of these genes, though this does not exclude modulation and additional contributions from post-transcriptional mechanisms. About a dozen *Saccharomyces* transcription factors have been causally associated with direct control of cell cycle expression patterns, including repressors, activators, co-regulators, and regulators that assume both repressing and activating roles, depending on context: Ace2, Fkh1, Fkh2, Mbp1, Mcm1, Ndd1, Stb1, Swi4, Swi5, Swi6, Yhp1, and Yox1. These can serve as internal control true-positive connections. Conversely, a majority of yeast genes have no cell cycle oscillatory expression, and true negatives can be drawn from this group. A practical consideration is how well the behavior of a network is represented in critical datasets. In this case, cells in all cell cycle phases are present in the mixed phase, exponentially growing yeast cultures used for the largest and most complete set of global protein:DNA interaction (ChIP/array) data so far assembled in functional genomics [21]. These data are further supported by three smaller studies of the same basic design [22–24]. This sets the cell cycle apart from many other transcription networks whose multiple states are either partly or entirely absent from the global ChIP data. Equally important are RNA expression data that finely parse the kinetic trajectory for every gene across the cycle of budding yeast [1,2] and also in the distantly related fission yeast, S. pombe [3–5]. This combination of highly time-resolved RNA expression data and phase-mixed (but nevertheless inclusive) ChIP/array data can be used to assign protein:DNA interactions to explicit cell cycle phases, while evolutionary comparison with S. pombe highlight exceptionally conserved and presumably fundamental network properties.

Many prior efforts to infer yeast transcription network connections from genome-wide data ([15–17,25,26] were designed to address the global problem of finding connection patterns across the entire yeast transcriptome by using very large and diverse collections of yeast RNA, DNA, and/or chromatin immunoprecipitation data. The present work focuses instead on a single cellular process and its underlying gene network, which represents a natural level of organization positioned between the single gene at one extreme and the entire interlocking community of networks that govern the entire cell. To model regulatory factor:target gene behavior, we adapted neural networks to integrate global expression and protein:DNA interaction data.

Artificial neural networks (ANNs) are structural computational models with a long history in pattern recognition [27]. A general reason for thinking ANNs could be effective for this task is that they have some natural similarities with transcription networks, including the ability to create nonlinear sparse interactions between transcriptional regulators and target genes. They have previously been applied to model relatively small gene circuits [28–30], though they have not, to our knowledge, been used for the problem of inferring network structure by integrating large-scale data. We reasoned that a simple single-layer ANN would be well-suited to capture and leverage two additional known characteristics of eukaryotic gene networks. First, factor binding in vivo varies over a continuum of values, as reflected in ChIP data, in vivo footprinting, binding site numbers and affinity ranges, and site mutation analyses. These quantitative differences can have biological significance to transcription output by affecting cooperativity, background “leaky expression” or the lack of it, and the temporal sequencing of gene induction as factors become available or disappear. This is quite different from a world in which binding is reduced to a simple two-state, present/absent call. Neural networks are able to use the full range of binding probabilities in the dataset. Second, ANNs can give weight and attention to structural features such as the persistent absence of specific factors from particular target groups of genes. This “negative image” information is potentially important and not used by other methods applied to date [15,21,31,32]. The inherent ability of ANNs to use these properties is a potential strength compared with algorithms that rest solely on positive evidence of factor:target binding or require discretization of binding measurements into a simplified bound/unbound call.

ANNs have been most famously used in machine learning as “black boxes” to perform classification tasks, in which the goal is to build a network based on a training dataset that will subsequently be used to perform similar classifications on new data of similar structure. In these classical ANN applications, the weights within the network are of no particular interest, as long as the trained network performs the desired classification task successfully when extrapolating to new data. ANNs are used here in a substantially different way, serving as structural models [33]. Specifically, we use simple feed-forward networks in which the results of interest are mainly in the weights and what they suggest about the importance of individual transcription factors or groups of factors for specifying particular expression outputs.

Here ANNs were trained to predict the RNA expression behavior of genes during a cdc28 synchronized cell cycle, based solely on transcription factor binding pattern, as measured by ChIP/array for 204 yeast factors determined in an exponentially growing culture [21]. The resulting ANN model is then interrogated to identify the most important regulator-to-target gene associations, as reflected by ANN weights. Ten of the twelve major known transcriptional regulators of cell cycle phase-specific expression ranked at the very top of the 204-regulator list in the model. The cell cycle ANNs were remarkably robust to a series of in silico “mutations,” in which binding data for a specific factor was eliminated and a new family of ANN models were generated. Additional doubly and triply “mutated” networks correctly identified epistasis relationships and redundancies in the biological network. This approach was also applied to two additional, independent cell cycle expression studies to illustrate generality across data platforms, and to probe how the networks might change under distinct modes of cell synchronization.

Analysis of the weights matrices from the resulting models shows that the neural nets take advantage of information about specifically disfavored or disallowed connections between factors and expression patterns, together with the expected positive connections (and weights) for other factors, to assign genes to their correct expression outputs. This led us to ask if there is a corresponding bias in the biological network against binding sites for specific factors in some expression families as suggested by the ANN. We found that this is the case, in multiple sensu stricto yeast genomes relatively closely related to *Saccharomyces cerevisiae,* and also in the distantly related fission yeast S. pombe. This appears to be a deeply conserved network architecture property, even though very few specific orthologous genes are involved.

## Results

**Figure 1:**
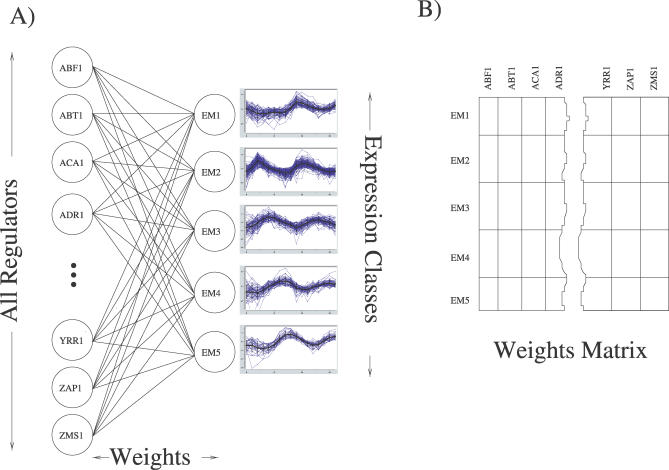
The Artificial Neural Network Architecture (A) Shown is the simple single layer network we trained to predict expression behavior based on the in vivo binding activity of ∼75% of the transcription regulators in yeast. A 204-dimension vector containing the measured transcription factor binding data from [21] was used as the input vector. Given this binding vector, the ANN was trained to predict which of the five cell cycle expression classes (clusters) each gene belongs to. These expression classes were determined using EM MoDG. (B) Matrix representation of the ANN. Each matrix cell, *W_c,r_,* represents the real-valued connection strength, or weight, between a regulator (*r*) and an expression class (*c*) and is shown in (A) as an edge between a regulator and an expression class. These weights represent the importance of binding activity or inactivity for each transcription factor in associating a member gene with its expression class (cluster) under the ANN model.

**Figure 2:**
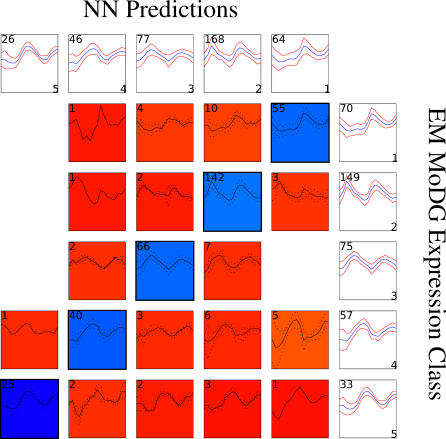
Confusion Array Display for the aobANN versus Membership in EM MoDG Expression Class Expression class predictions from the aobANN (based on ChIPchip factor binding data) are displayed in a confusion array against the starting expression classes from EMDoG clustering. Each of the 40 contributing “best” ANNs were trained on 80% of the data and tested on the remaining 20% to evaluate performance. They were selected as the best performing network out ten networks trained on the same data split, but initialized with differing random seeds. These two classifications have a similarity of .86 by linear assignment [9]: an LA value of 1.0 would indicate perfect classification success by the ANNs.

Classifier ANNs were trained to predict membership in cell cycle phase-specific RNA clusters, based on global transcription factor binding data (Figure 1). As expression input data, these ANNs used time course microarray data [2] for 384 cycling genes that had been grouped into five clusters by an expectation maximization (EM) algorithm [9]. As measured by receiver operator characteristic (ROC) analysis, these clusters are quantitatively well-separated from each other, with less than 10% overlap at their margins with any other clusters, except that the S-phase cluster (EM3) was somewhat less well-separated from its kinetic neighbors, EM2 and EM4 [9]. The primary goal of the ANN modeling is to infer the set of regulatory connections that underlies each of the cell cycle–phased expression groups. Note that a given cluster might be composed of more than one regulatory subgroup; it need not be the case that all associated regulators interact with all–or even most–of the genes in a cluster. ANNs were trained to assign expression cluster membership for each gene based on 204 measured binding probabilities from ChIP/array experiments ([21]). To accommodate the scarcity of data, while minimizing effects of overtraining, we generated an average-of-bests artificial neural network (aobANN) (Methods). As anticipated, the aobANN classified input genes best, correctly assigning the expression class of 86% of included cell cycle genes (Figure 2). Individual best-of-ten networks, each trained on 80% of the data and tested on the remaining 20% correctly assigned expression class membership for ∼50% of the genes, with an accuracy range between 40% and 65%, whereas only 27% of genes would be expected to be classified correctly if genes were classified by a random process (Figure S1). As shown in Figure S2, a substantial fraction of genes (32%) are always classified correctly by every ANN, another subset (28%) are never classified “correctly,” and the remaining fraction (40%) are intermediate. An examination of possible correlates of high or low predictability, including absolute level of RNA expression and bidirectional versus unidirectional orientation of the gene relative to its upstream neighbor found no correlation except that the EM2 (late G1) class is enriched in highly predictable genes, while the EM5 (M phase expression peak) is most impoverished (Figure S2). The major conclusion from global statistics is that individual ANNs and the aobANN have developed weighting schemes that are effective in connecting factor binding information from ChIP/array to RNA expression patterns, even in the presence of considerable experimental noise that is a widely acknowledged property of the input datasets.

### Parsing the ANN Weight Matrix to Infer Regulatory Relationships

**Figure 3:**
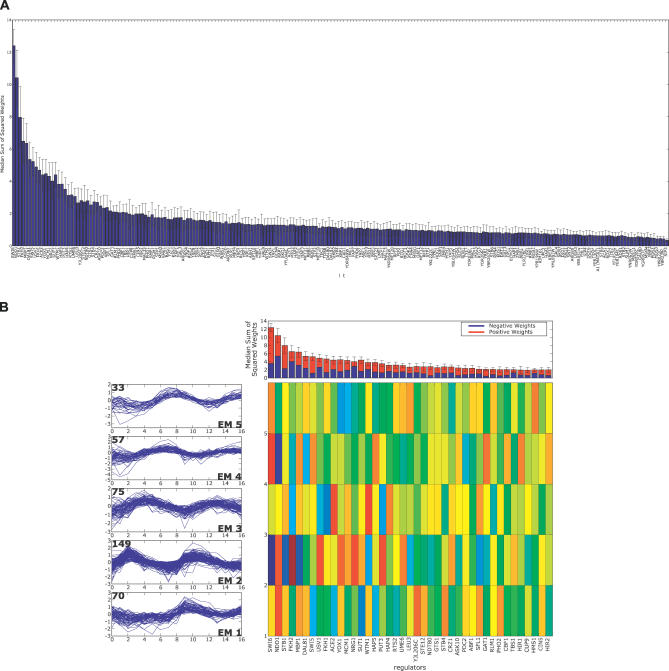
Weight Matrix Analysis for the aobANN (A) Regulators were sorted based on the SOS metric (Methods and text), and the resulting total SOS rank for each regulator is plotted as a bar. (B) The top 20 regulators are shown, ordered by importance in predicting expression behavior using the sum-of-squared weights metric. The top panel reproduces a zoomed-in view of the top 20 regulators as in (A). The bar representing each regulator is split to display positive (red) and negative (blue) contributions. The left-hand column shows a trajectory summary for each expression cluster as classified by EM MoDG. The right-hand side color map represents the weight matrix where expression classes are displayed along the rows corresponding to the drawn trajectory summaries. Regulators are sorted along the columns in rank order. Each cell is colored according to its value in the weights matrix.

**Figure 4:**
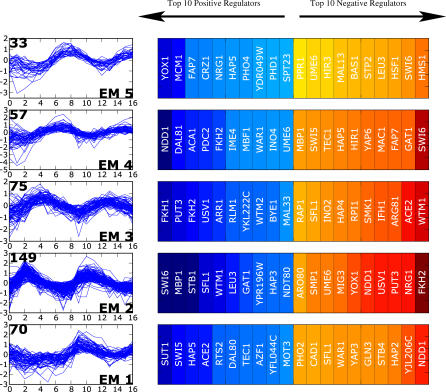
ANN Weights Sorted According to Expression Class ANN weights from the aob network for the top-ranking and bottom-ranking (high negative weights) for each class. The regulator ranking for each class is determined by its value in the aobANN weights matrix for each expression class. Detailed annotations for these regulators are given in Table 1.

We next interrogated the aobANN weight matrix to find out which regulators are most important for assigning genes to specific gene expression behavior. Regulators were sorted by a sum-of-squares (SOS) rank calculation (see Methods) over the expression classes. The factor ranking, based exclusively on the ANN weights, assigned nearly all transcription factors previously definitively associated with phase-specific regulation to the very top of the ordered list. Figures 3 and 4 summarize data from the weight matrix of the aob network. A plot of the sum of squared weights for each factor shows that the top 10% of all regulators carry much higher weights than all the rest, and the dropoff in weight is quite dramatic (Figure 3A). Focusing on the top 20%, the relative contribution to each sum derived from positive (blue) versus negative (red) weights is shown (Figure 3B). Both negative and positive weights contribute substantially, and the way in which weights associate with each individual expression class is shown in Figure 3B. The top regulators in this ranking are Swi6, Ndd1, Stb1, Fkh2, and Mbp1, all of which are known direct regulators of the cell cycle. In most instances high positive weight for a factor (blue) is associated with the expression class or pair of classes expected from more detailed molecular genetics studies. For instance, Swi6, Stb1, and Mbp1 are the first, second, and sixth ranked regulators, and they are known to function together at genes expressed in EM2 (G1). Mbp1 binds DNA directly, and Swi6 and Stb1 bind to Mbp1 [34,35]. Ndd1 and Fkh2, the second and fourth ranked regulators, also function together in a molecular complex [36]. In the aobANN model, they are associated with EM3/4 (S/G2), again recapitulating expected domain of action.

**Table 1:**
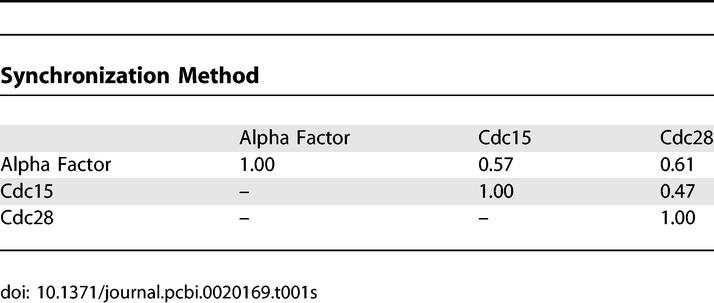
Similarity of Clustering Results from Different Synchronization Methods as Measured by Linear Assignment [9]

### ANN Stability

**Figure 5:**
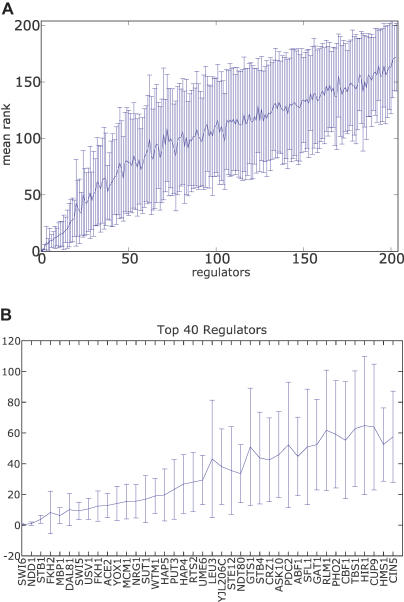
Neural Network Rank Order Stability (A) Regulators are sorted by their SOS rank order (see text and Methods). The line indicates the mean rank for each regulator across each of 40 best ANNs, with variance of each ranking indicated by the error bar. (B) Top 20 regulators show high stability across ANNs.

Regulator-to-target relationships suggested by the ANNs were very stable with respect to permutation of the input DNA binding data and to a range of biologically reasonable differences among input expression clusterings (classifications). We find the relative ranking of the top regulators to be stable across all networks generated during the training paradigm (Figure 5). The ranking of regulators was also stable across networks that were trained to predict expression classes derived from clusterings with either more or fewer clusters (the experiment was performed over K = 4, 5, 6, 7, or 8, and results are summarized in Figure S4). Lower K values than 4 fit the data poorly and are therefore irrelevant; and still higher K values above 8 force an entirely unjustified oversplitting of clusters that is clearly inappropriate.

### In Silico Network Mutations

**Figure 6:**
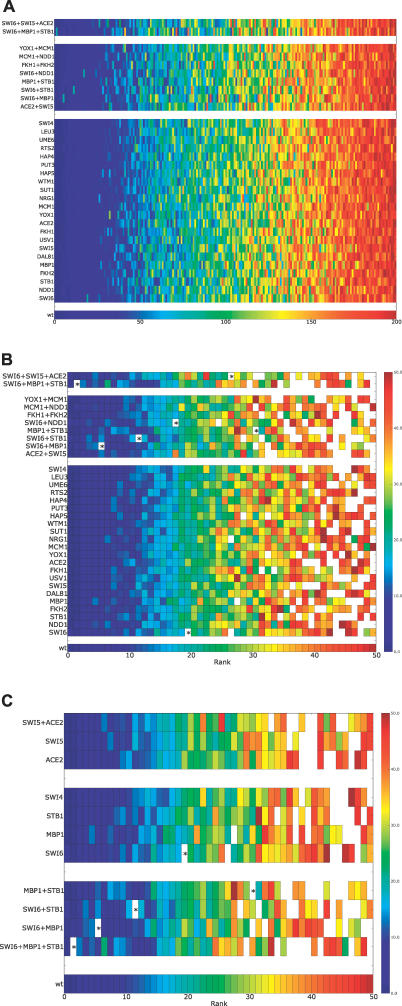
In Silico Network Mutations Shown are results from training ANNs missing one or more regulators as indicated on the left margin of each heatmap. Within each heatmap, each cell represents a regulator, the position of the cell along the *x*-axis of the plot is determined by the mutated network, but the color is indicative of the regulator's rank in the unperturbed network (as shown in Figure 3). The lowest strip shows the rank order color spectrum for the wild-type network. (A) An overview showing the overall rank stability of the regulators across all mutant networks generated. (B) A higher resolution view of the top-ranked regulators for each mutant network. Only the top 50 regulators are shown, and the color spectrum is adjusted to only span 1–50. Any regulator that was ranked within the top 50 regulators in a mutant network, but not in the wild-type network, is shown as white. The position of Swi4 in each network is denoted by *. (C) A zoomed-in version of our mutant network analysis focusing only on networks generated by the top G1 regulators (Swi6, Mbp1, Stb1, Ace2, Swi5, Swi4).

We next performed a series of in silico network mutations in which binding data for one, two, or three top-ranked regulators were removed before training a new set of ANNs. The resulting deletion ANNs were used to produce a new aob network, as before, and the corresponding sum of squared weights ranking was constructed (Figure 6). These perturbations further test network stability and also identify specific instances of factor redundancy. Overall the ANNs proved remarkably stable to elimination of high-ranking factors. When each of the top 20 were eliminated singly, the identity of the remaining top regulators proved very stable (Figure 6A). The color code for each cell reflects its rank order from the parental, unperturbed network (shown in the bottom row). Each subsequent row reports the outcome for the mutant network with the indicated factor or factors removed. Although the cells are placed according to their rank order in the mutant AOB network, the color is based on the ranking from the unperturbed, “wild-type” network. In general, factors from lower rankings were not promoted into the high ranking (dark blue) domain, nor were previously highly ranked factors (blue) demoted significantly into yellow and red domains. Thus, the first major conclusion from the mutation experiments is that neither the connections the ANNs infer nor the absolute performance of the ANNs depends heavily on a single factor or even a factor pair. The ability of the models to highlight other important connections is not compromised by elimination of any high-scoring factor.

Figure 6B shows the same mutant networks at higher resolution, so that all factors whose original rank was >50 appear in the summary as white cells. Original rank order is again indicated by the color of each cell, although the color scale has been shifted to make it more sensitive to changes in rank among the top 50 regulators. A few specific exceptions to overall stability were observed, in which a relatively low-ranked regulator has been elevated by mutation into higher ranks. The most striking example is Swi4, which is demarcated with a star. Swi4 is a very well-studied cell cycle transcription factor that did not fall in the top 10% in the wild-type network (it ranked 80th). As shown in Figure 6C, “mutant” networks for all factors associated with the G1 (EM2) caused Swi4 to advance in rank, with double or triple mutations moving it progressively higher. We discuss later the causes and consequences of Swi4′s initial low ranking in the wild-type ANN and the implications for detecting biological redundancy. However, the general conclusion for ANN analysis is that systematic single and multiple perturbations of high-ranking regulators provide a way to detect redundancy, even when a connection—here Swi4 with G1—was not evident in the unperturbed wild-type ANN. Additional double and triple mutations for the major cycle classes were performed and no other change as remarkable as Swi4 was found.

### Out-of-Sample Accuracy

We next tested out-of-sample accuracy, which is the ability of the training paradigm to generalize to another set of independently collected binding measurements, in which both experimental error and biological error will differ from the first series of models. We constructed a new aobANN trained again from data collected from Harbison, but included only binding measurements from the 111 regulators available in both the Harbison et al. (2004) study and the independent Lee et al. (2002) study. Despite biological and experimental difference between the two datasets, this aobANN delivered a highly significant out-of-sample accuracy of 56%, which is 17 standard deviations from the average linear assignment score (.27 ± 0.017) of a random partitioning of the genes, where class sizes are determined by drawing from a multinomial distribution based on the cluster sizes.

### Regulator Rank Stability and Power

The stability of weight ranks across the 40 individual “best” networks that contribute to the aobANN was examined. We postulated that factors whose rankings are less stable across many individual networks would also be less likely to be functionally significant than factors showing high stability across the individual networks, even if the median SOS weight is quite high in all cases. The well-known regulators of cell cycle transcription, ranking in the top dozen, showed greatest stability, and a substantial discontinuity was found to separate the top 20 from the remaining factors (Figure 5). We then asked how well the top regulators can perform if they are used to build a new aobANN over a sweep that ranges from three to 28 regulators. This experiment showed that a network built from the top 20 regulators performed almost as well as the full 204-regulator network and ranked its regulators very similarly (Figure S3). The top five regulators on their own (Swi6/Mbp1/Stb1 plus Fkh2 and Ndd1) were surprisingly powerful in parsing G1 versus G2/M. Conversely, an aobANN composed from the bottom 184 regulators was much less successful in predicting expression.

### ANN Models from Independent Cell Cycle Experiments

We next independently clustered Cdc15 TS and alpha factor synchronized cell cycle RNA expression data [1], and used these new clusters to build two new ANN cell cycle models. These datasets are from two different cell cycle experiments, each measured using deposition microarrays and a ratiometric design, in contrast to the cdc28 arrest described above, which used Affymetrix data. By focusing on each synchronization method individually, rather than using a merged dataset, we aimed to capture possible differences in the biology that might arise from different methods of synchronization, while also revealing the relationships that are robust across the three experiments and two assay platforms. The ChIP/chip dataset is unique and was therefore used to build ANNs across cdc28, cdc15, and alpha factor experiments.

**Figure 7:**
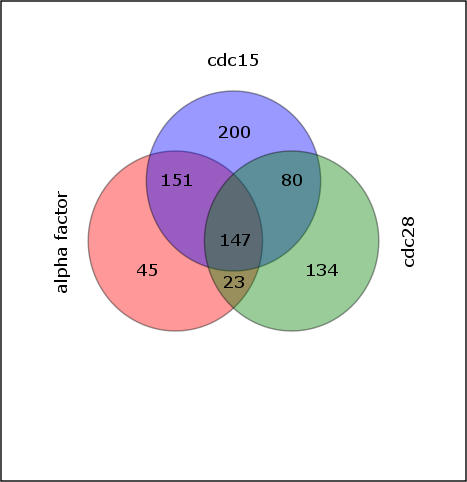
Overlap of Cell Cycle Groups Venn Diagram for the total number of genes cycling in each of the three synchronization methods after our filtering and normalization.

As demonstrated with the cdc28 data above, we found these additional ANN models return the same core cell cycle regulators highlighted by the cdc28 ANNs. Six of these; Ndd1, Mbp1, Swi5, Stb1, Swi6, and Fkh2 are among the top seven regulators found, regardless of which cell cycle data and clusterings were used as input to the ANNs. This robustness in the central regulatory relationships is quite remarkable considering that, of 780 genes belonging to at least one of the cycling datasets, only 147 genes are common to all three experiments. Quantitation of pairwise clustering overlap, using the linear assignment metric, makes it very clear that the gene number and clustering patterns differ substantially (Figure 7). Thus, ANNs highlight major shared cell cycle relationships, even though the gene sets used and the clusterings are quite different (Table 1).

**Figure 8:**
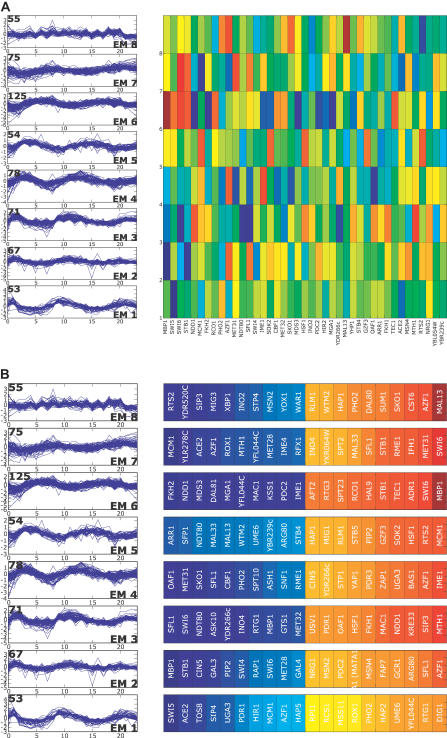
Transcription Factor Rankings by aobANN Weights for Cdc15 Syncrhonized Data (A) ANN weights are sorted by the SOS metric as in Figure 3B. (B) ANN weights from the aob network as in Figure 4 for ANNs trained to predict RNA expression clusters derived from yeast cultures synchronized using Cdc15 TS mutant [1].

**Figure 9:**
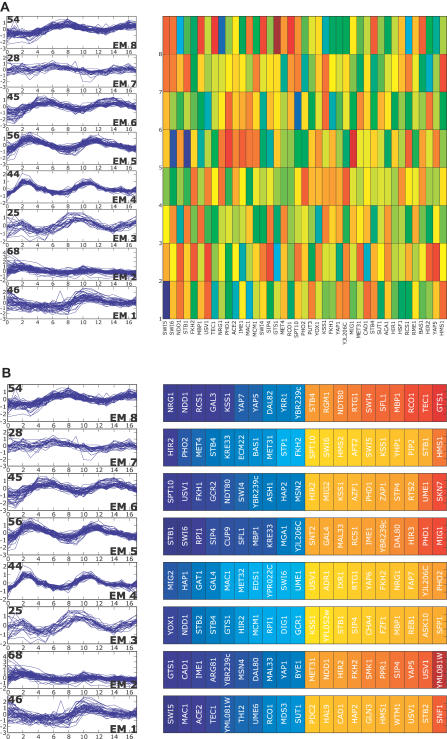
Transcription Factor Rankings by aobANN Weights for Alpha Factor Arrest Data (A) ANN weights are sorted by the SOS metric described in the text and in Figure 3B. (B) ANN weights from the aobANN network, as in Figure 4, for ANNs trained to predict RNA expression clusters derived from yeast cultures synchronized using alpha factor arrest to syncrhonize cells [1].

Cdc15ts-synchronized cells are arrested at the end of M phase [1]. Correspondingly, we find the expression cluster that peaks first—at 10 min in the Cdc15 data—associates strongly with the early G1 factors Swi5 and Ace2 (EM1 in Figure 7). Note that in the previous cdc28 ANN, the same association was made, even though—under that release condition—genes of this regulatory group are not upregulated until the second cycle after release [9] and above). Alpha factor arrest is similar in this way to cdc28, reflecting their similar blockade points. Thus, the ANNs easily related the cdc15 early G1 cluster to the alpha factor and cdc28 early G1 clusters, even though the cluster trajectory is strikingly different and the clusters themselves contain no individual genes in common with the cdc28 or alpha factor datasets (Figures 4, 8, and 9). Other high-ranking regulators appear in one or two, but not all three ANN cell cycle models. Yox1 and Yhp1, for example, differ among the models, because the gene classes derived from the RNA clusterings differ in content. Finally, Pho2 emerges as a potentially significant regulator associated with an M-phase kinetic pattern in the two Spellman datasets, consistent with the previously reported Pho2/Pho4 mediated, cell cycle expression for some phosphate-regulated genes [37]. This is thought to be due to intracellular polyphosphate pools, which vary through the cycle in some culture conditions, but can also be influenced by growth media and history.

##  Discussion

We found that single-layer ANN classifier models can effectively integrate global RNA expression and protein:DNA interaction data (ChIP/chip). The resulting models prominently highlight factors known to drive the transcriptional regulatory network underlying cell cycle phase-specific expression. The weight matrices from these ANN models generally associated previously known cell cycle transcription factors with the cell cycle phase they are thought to regulate, and they did so as well as or better than other methods, based on flexible iterative thresholding [15], network dynamics [16], or, most recently, Bayesian methods [31]. In general, we feel that more conventional statistical approaches and ANNs complement each other. Both generate hypothesized relationships and rank them. The strength of the single layer neural network architecture used here is that it mirrors several basic properties of natural gene networks: 1) both presence and absence of factor binding determine when and where a gene is expressed; 2) factor occupancy in vivo is a continuum, not an all-or-nothing phenomenon, and the graded differences can have biological significance. For example, graded binding of the transcription factor Pha4 creates spatiotemporal gradients of target gene expression during pharyngeal development in C. elegans [38]. These features of the neural network distinguish it from algorithms that depend solely on positive evidence of binding and require discretization of the binding signal to bound or unbound. A further distinction is that the neural network models can be easily and informatively “mutated” to ask how the overall network connection patterns and outputs are affected by specific changes, such as eliminating data for individual factors, combinations of factors, or making even larger structural changes. The obvious complementary strength of statistical methods is in quantitative thresholding based on significance measures.

A general conclusion that can be drawn from this work comes from the overall success of ANNs in classifying expression output according to transcription factor binding patterns. This might not have been true, but this overall observation argues strongly that transcriptional regulation, rather than differential post-transcriptional regulation, is the dominant mechanism in shaping phase-specific RNA prevalence clusters. This observation does not preclude a role for other mechanisms operating on a minority of genes (perhaps explaining some difficult-to-predict genes) or a post-transcriptional role that is uniform over an entire class. For example, confusion matrix analysis of expression classes versus the predicted expression pattern from the ANNs identified a group of genes with EM3 (S phase) kinetics that comprise 10% of that cluster, but are associated with the EM2 G1 group by the ANN model (Figure 2), and these are reasonable candidates to be differentially regulated by post-transcriptional processes such as slower turnover.

### Relating the Inferred Connections to Known Biology

The sum-of-squared weights metric proved to be simple and useful for objectively ranking regulators according to their importance in the network model, regardless of the input expression dataset. Even though ANN weights are not direct physical measures of binding, the resulting rankings correspond remarkably well with what is known from decades of work on transcription in the yeast cell cycle. The ANN models even highlighted subtle regulatory differences between different cell cycle synchronization methods. The top dozen of the 204 total regulators in the cdc28 ANN model contained ten of 12 transcription factors present in the Harbison ChIP dataset and are known to operate on cycling genes. Swi6 ranked at the top of the cell cycle regulators list in the cdc28, cdc15, and alpha factor ANN models, and is always associated with G1 expression. Swi6 also shows a relative absence of binding to genes highly expressed during G2. The pattern of weights evaluated across the RNA expression clusters provides additional information. For instance, the cdc28 ANN weight vector for Mbp1 across the cell cycle clusters tracks very closely with Swi6 (correlation coefficient r = .92). This mirrors underlying molecular biology in which Mbp1 and Swi6 combine to form the heteromeric active G1 transcription factor MBF. Stb1 is similarly grouped with Swi6 and Mbp1 as a co-regulator of G1 (cdc28 EM2) genes (r = .95 and .89 for Stb1 with Mbp1 or with Swi6, respectively). Ace2 and Swi5 are paralogous factors with similar DNA binding target sites [39,40], and both are positively associated with the early G1 (cdc28 EM 1) expression profile with similar in-weights profiles (r = .71).

Also confirming expectations from studies of target genes and epistatsis predictions, Fkh1 and Fkh2 were associated with cdc28 S/G2 expression clusters by the ANN. This implies that joint association is consistent with double knockout experiments, which indicate that the two complement each other [41], and with studies showing the two factors bind the same sites in vitro [42]. Examined in detail, the cdc28 ANN weights suggest a more nuanced view, in which both Fkh1 and Fkh2 are important for some genes in early S/G2 (EM3), whereas S/G2 class genes (cluster EM4) rely more heavily on Ndd1 and Fkh2 and less on Fkh1. RNA expression data for Fkh1 and Fkh2 is consistent with this, since Fkh1 increases in expression nearly 20 min before Fkh2, in expression data collected by Cho et al. in 1998 [2]. This is also consistent with a detailed study of in vivo binding at a few specific target genes [42], which showed that the two Fkh factors do not bind identically in vivo, and that there is a distinction between genes of the so-called Clb2 cluster (a subset of Cluster EM4 here), that are dominated by Fkh2 in conjunction with Mcm1/Ndd1, versus Fkh1, which is thought to bind independently. The alpha factor and cdc15 ANNs place diminished emphasis on Fkh1, compared with cdc28 ANNs, which is consistent with the idea that the two factors have different molecular activities and targets.

#### Time and sign of action.

Cdc28 ANN weight vectors for Mcm1 and Yox1 were also correlated (r = .69), defining an association with EM5 target genes where they displayed the two highest positive weights. They are known to act on some of the same genes, including EM5 group members [43]. In this example, the ANN is picking up molecular effects that are of opposing molecular activity, with Yox1 repressing Mcm1 activity. This illustrates an issue of interpretation. Because the original binding data are from a mixed phase cell population, it reveals nothing about when during the cycle detected binding occurs. For positive acting factors whose binding and function are contemporaneous, we see a peak of binding simply correlated with a peak of RNA expression. But for a repressor acting on genes expressed in M phase, binding occurs at other times (late G1, S, G2 alone, or in combinations [43]). Thus, the ANN correctly connected the factor with its targets; but only by independently determining the mode of Yox1 action, or by adding temporally resolved binding data, can the sign and timing of action be discerned. For factors whose action—repressing or activating—is unknown or is conditional depending on context, temporally resolved ChIP data will be needed to infer the mode and time of action.

#### Swi4, a “missing” regulator.

The ANN models did not assign high weight to Swi4, which one would expect to rank highly. Although Swi4 is a well-known direct transcriptional regulator of Early G1 genes, providing the DNA binding moiety of SBF factor [44], it was not even close to the top 20 in the cdc28 aobANN, ranking 80 of 204. Its preferential association with G1 target genes only came to light when we performed in silico mutation analyses, eliminating one or more G1 factors. There are two possible explanations for its weak values in the wild-type ANNs, and they are not mutually exclusive. One simple possibility is that redundancy with other G1 regulatory factors is widespread, and this masks Swi4 when training the ANNs. Especially if coupled with generally less robust signals in the ChIP assay, the ANNs might have simply ignored Swi4. A second explanation is that Swi4 has greater breadth of binding across multiple clusters than its paralog, Mbp1. In this scenario, Swi4 spills over, binding to members of multiple cell cycle expression clusters when compared with other G1-specific regulators such as Mbp1, Swi6, or Stb1. This would give Swi4 less discrimination power in classifying genes, despite active G1 binding and could arise from purely technical issues, or from an unappreciated biological role outside its function in SBF.

An independent analysis of the Harbison ChIP data in the context of a much larger library of expression data across many conditions other than cell cycle phases, using a different computational approach, supports the idea of broad Swi4 distribution among cell cycle regulatory classes [15]. Specifically, the GRAM algorithm uses coexpression patterns to incorporate into the connection map ChIP interactions that are below statistical significance when evaluated on their own [15,21,24]. They reported regulatory modules consisting of pairs of factors in which Swi4 is partnered by binding and expression data with one or more factors from each and every expression cluster: Ace2, Fhk2, Ndd1, and Mcm1, as well as the “classic” associated G1 factors, Mbp1, Stb1, and Swi6. In addition, an entirely independent set of ChIP/chip measurements and analysis from Snyder and colleagues [22] showed substantial Swi4 binding activity upstream of non-G1 genes. Taken together, these data suggest Swi4 might have one or more previously unappreciated functions within exponentially growing cells that are distinct from its classic role as part of SBF.

Finally, a picture of partly, but not entirely, redundant functions for the Swi4/Mbp1 paralogs was also emphasized in a recent genetic study [45]. We therefore think it likely that the way the unperturbed ANNs treat Swi4 reflects partial biological redundancy combined with its more widely distributed binding across non-G1 clusters.

### Potential Newly Identified Regulatory Connections

Do the ANNs suggest new factors associated with phase-specific expression? Focusing on the cdc28 example, and using stability across ANNs as an added filtering criterion, factors ranking above Leu3 stood out. In particular, both Usv1 and Dal81 are interdigitated among the otherwise well-documented ten major cell cycle regulators, although not previously associated with this function to our knowledge. A different explanation is that factors such as Usv1, Dal81, and a handful of others ranking in the top 20, may be in the ANN model for reasons having nothing to do with the cell cycle explicitly, but having much to do with the partially overlapping architecture of transcriptional networks in eukaryotes. Thus, we expect that some genes—perhaps most—within cycling clusters will also belong to one or more other functional modules. In the context of those other functions, they will presumably be regulated by factors that have nothing to do with directing cell cycle phase patterns. This kind of network intersection and partial overlap is strikingly evident in global module maps [25]. Some factors appearing in the ANN top 20 may be there for this reason. There are others (Pho2, for example) that seem to be drawn into regulating phase-specific expression because of metabolic links (in this case through polyphosphate pools and membrane biogenesis [37]). We expect that the overall approach we have taken for the cell cycle network, using global ChIP/chip data, could easily be extended to any network whose states of interest are well-represented in available ChIP/chip data, and whose RNA datasets are of sufficient quality and resolution to cluster the expression behaviors of interest. However, a decisive improvement in sophistication of the ANN model, and the hypotheses it generates, will come with time-resolved ChIP data.

### Neural Network Weights Predict Evolutionarily Conserved Binding Motif Frequencies

**Figure 10:**
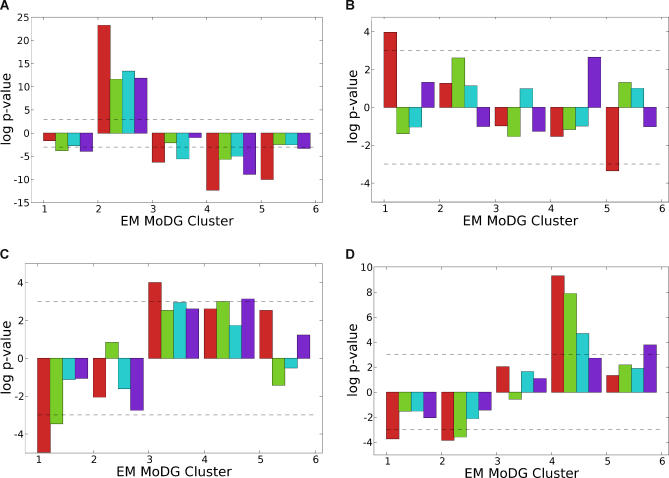
Enrichment and Depletion of Binding Sites in Individual Cell Cycle Phase Classes for Transcription Factors Highly Ranked in aobANNs in Budding Yeast Genomes For several regulators highlighted by strong positive or negative association with particular expression classes in Figure 4 (denoted parenthetically), site enrichment *p*-values were calculated for each EM MoDG expression cluster. Each *p*-value was calculated using only the cell cycle identified genes that were also used as input genes to the ANN. Each block of bars along the *x*-axis represent log *p*-values (*y*-axis) for an EM MoDG cluster. Each bar within these blocks represents the log *p*-value measurements for a different *Saccharomyces* species as indicated by the color legend. Enrichment is shown as positive values (−log *p*-value), and depletion is shown as negative values (log *p*-value). The species have been arranged by to reflect evolutionary distance from S. cerevisiae. From left to right: *S. cerevisiae, S. paradoxus, S. mikatae, S. bayanus*. A dashed line along the graphs at *p*-value = .05 has been drawn to help visualize the scale difference between the plots. (A–D) Enrichment bar charts for the specified binding sites. If the binding site is referred to by a standard name other than that of the regulator that binds to it, the regulator name is in parentheses. The color map key for each specie is at the bottom.

If binding data are predictive of expression class, and if meaningful transcription factor binding is motif-specific, then it should be possible to independently verify relationships from the weights matrix by measuring the frequency of binding motifs. We can also ask if any observed site enrichment and depletion are evolutionarily conserved, as would be expected if they mediate functionally relevant factor binding. Motif frequency across cell cycle clusters in multiple yeast species correlated remarkably well with binding probabilities from the ChIP data and also with the ANN weights trajectories across the same clusters (Figure 10). The conserved motif data for Mbp1 and Swi5/Ace2, and Fkh1/Fkh2, all factors with well-defined binding motifs, provided independent support for conclusions from the ANN, since the ANN was constructed without any input information about DNA sites.

### Conservation of Site Enrichment and Depletion over Great Evolutionary Distance

**Figure 11:**
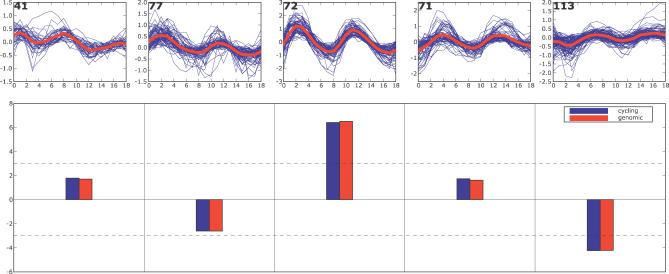
Binding Site Enrichment and Depletion for *S. Pombe* MCB consensus binding site enrichment *p*-values are shown for *S. pombe,* based an EM MoDG clustering of expression data from ([3]. Cluster trajectory summaries as a function of timepoint in the cell cycle are shown for each expression cluster in the top panels; red lines highlight the mean expression trajectory, and cluster gene number is given in the upper left corner. Below is a bar chart of *p*-values. *p*-Values are normalized against only cycling genes (blue), or are normalized against all genes (red).

The distribution of MCB sites across the cycle phases was striking and prompted us to ask if both enrichment and depletion holds over very great evolutionary distance. If specific depletion is a functionally important network characteristic, then we would predict that it would be retained over very great evolutionary distance. We performed the same site enrichment analysis across cell cycle gene classes in *S. pombe,* which is said to be as distant from budding yeast as are humans (∼500 my). We used the EM algorithm to cluster the S. pombe cycling data of [3] in the same way that the various S*accharomyces* experiments had been clustered [9]. At this evolutionary distance there are no large blocks of conserved noncoding DNA sequence. S. pombe does, however, have an identified MBF ortholog, and the short binding motif for MBF shows significant site enrichment in our expression cluster 3, together with significant depletion from cluster 5, mirroring the pattern in budding yeast (Figure 11). The positive regulator-to-target group conservation was noted previously [3–5], but in this study we were able to detect it without strongly prefiltering gene sets for their explicit experimental responsiveness to MBF. The new observation here is that depletion of MBF sites, operating specifically in the group of genes normally expressed later in the cell cycle, is a very highly conserved property. This cis-motif depletion suggests there is selective restriction against MBF sites and that it is phase-specific: it does not apply broadly to most genes in the genome, but does apply preferentially to genes in late cell cycle cluster (in this case cluster 5 for *S. pombe,* cluster 4 for S. cerevisae). In both organisms, this cluster contains genes whose products are involved in mitosis, and it seems possible that their heterochronic expression during G1/S phases, as MCB sites might cause, could disrupt proper control or execution of S phase. However, the observed conservation is apparently a network property, even though the specific genes in each phase group are—mainly—not orthologous. Thus, the surprising observation that most genes in these oscillating clusters are not the same ones in *pombe* and *Saccharomyces* (reviewed in [13]), if correct, suggests that conserved enrichment and depletion of regulatory motifs are network architecture properties that are shared across hundreds of millions of years, even though most specific genes involved are different.

### Materials and Methods

#### Data pre-processing.

The primary expression dataset for modeling is Affymetrix microarray data measuring RNA levels of nearly every gene in yeast through two cell cycles, following release from conditional CDC28TS arrest [2]. That time course sampled RNA levels at 10-min intervals over 170 min, which covers two cycles. These data were obtained from the original authors and preprocessed in three steps. 1) Any gene that did not show sustained absolute expression greater than the 2.5% quantile of the data (an absolute signal of 8) for three consecutive timepoints was eliminated. 2) For the remaining 6,174 expression vectors, each time point measurement was divided by the median expression value across all time points for the gene. 3) The log2 of each ratio was then taken, and these values comprised the expression matrix for all further analysis. For key model building in this work, we focused on the subset of expression vectors (384) that had been identified by Cho et al. as displaying a cell cycle dependent pattern and also passed the above filter for absolute expression; operationally we refer to this set as the “cycling” set.

The primary in vivo protein:DNA interaction dataset (ChIP/array) used here is from [21]. These data were obtained at http://web.wi.mit.edu/young/regulatory_code/ and the reported *p*-values were used directly. Briefly, for each of 204 transcriptional regulators, Harbison and colleagues constructed a yeast strain containing a myc-epitope-tagged version of the factor that was inserted into the corresponding transcription factor locus. Each strain was used to perform three independent ChIP/array measurements taken from freely cycling exponential phase cultures. The cells were subjected to standard formaldehyde crosslinking to attach transcription factors to their in vivo binding sites, the chromatin was sheared, factor-bound DNA was enriched by IP, amplified by LMPCR, and fluorescently labeled. ChIP-enriched DNA was then co-hybridized with control DNA to microarrays containing essentially all intergenic sequences in yeast. A binding ratio was then calculated for each array feature based on the relative hybridization signal for targets synthesized from ChIP enriched DNA versus whole cell extract control DNA. Three biological replicate experiments were performed, each beginning from an independent yeast culture. Based on an error model first described in [46] and the three replicate binding ratios for each intergenic sequence, a *p*-value was reported for each upstream intergenic sequence. This *p*-value roughly estimates the probability that a given transcription factor is bound to a particular intergenic sequence.

#### Neural network implementation and training.

Figure 1 illustrates the overall structure of the ANN trained in this study. Backpropagation was implemented by the UWBP package [47] to train a single layer network with no hidden units. Each ANN was trained using 300 epochs using a learning rate of .002. RNA expression array data for the subset of 384 cycling genes as described above were clustered using an expectation maximization algorithm fitting the data to a mixture of Gaussian probability distributions with diagonal covariances (EM MoDG [9,48]). Networks to predict cluster membership for each gene based on an input vector composed of ChIP derived in vivo factor binding probabilities for the 204 measured regulators in the Harbison dataset. Individual networks were trained using 80% of the data and tested on 20% of the data. For each 80/20 dataset split, ten neural networks were trained using different random seeds for each network. The network with the best prediction accuracy on the testing dataset was then selected and denoted as “best.” This process was then repeated 40 times, splitting the dataset into different testing and training datasets. The network weights from the resulting 40 selected “best” networks were then averaged together to create the aobANN. We focus on this network for subsequent biological interpretation, with the primary goal of identifying regulatory connections between transcription factors and their direct target genes. Because the purpose of this network is not to repeatedly classify similar data, the implications of overtraining are different than they would be for classical uses of ANNs. In this unconventional usage, we show by measuring the behavior of ten internal “gold standard” known cell cycle regulators, that any “overtraining” is not deleterious for the intended goal, which is extracting a series of ranked hypotheses about regulator-to-output relationships. Regulators within aobANNs are ranked based on the median SOS rank across all the individual ANNs trained to generate the aobANN. The SOS ranking for a regulator within an individual network is simply the sum of squared weights across the classes in the weight matrix (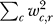 ).

#### Consensus site enrichment and depletion calculations.

To determine whether an expression cluster showed an enrichment in genes that contain a particular consensus site, we calculated the likelihood of the observed enrichment, or depletion, being a chance occurrence according to a binomial model of occurrence probabilities. We count the observed number of genes that have at least one instance of a consensus sequence within the 1 KB directly upstream of the coding sequence for all genes in an expression cluster versus the number of genes that would be expected by chance. As no known background sequence model is completely provably correct, for each consensus sequence we calculate the expected background frequency ( *f̂*) using a bootstrapping method. We randomly selected 1,000 different sets of genes the same size as the cluster being compared (*n*). These randomly selected background sets are drawn from either the entire genome or from only the “cycling” genes, which were used in training the ANNs. The number of genes that contain at least a single instance of the consensus is counted for each randomly selected set. The average count across the 1,000 samples is normalized and used as our estimate of the expected number of genes within a cluster that have a single occurrence within 1 KB upstream (*E_C_*). Since the chances of any given gene within a cluster having a given consensus sequence within the 1 KB upstream can be assumed to be independent, we can estimate the probability of finding the observed number of counts (*O_C_*) using a standard binomial distribution (Equation 1). If the site is enriched, we estimate the *p*-value for the likelihood of finding at least the observed count, but if the site is depleted we calculate likelihood of finding at most the observed count (Equation 2).

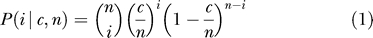


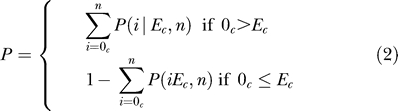



## Supporting Information

Figure S1 ANN Prediction Accuracy Histogram and Correlations with Binding and Expression Levels We trained 40 ANNs (see Methods) to predict a gene expression behavior from only the regulator binding activity upstream to its start of transcription. For each network, we trained on 80% of the data and tested on the remaining 20%.
(A) The distribution of ANN accuracy across the 40 trained ANNs. Along the *x*-axis are bins of accuracy ranges, the *y*-axis counts the number of ANNs that showed the designated prediction accuracy.
(B) Displays the relative reproducibility of the ANN rankings. Each regulator was ranked by its net influence in the ANN using a sum of squared weights metric across the classes in the weight matrix. Shown is a scatterplot of the regulator ranks from the first 20 ANNs versus the second 20 ANNs trained.
(C) Scatterplot of the predictability (fraction of ANNs correctly classifying a gene correctly) versus mean absolute expression level of the four highest measured time points for each gene.
(D) Predictability versus mean binding level for the ten highest bound regulators.
(154 KB PDF)

Figure S2 Distribution of Neural Network Prediction Accuracy across EM MoDG Expression Pattern Clusters The *y*-axis on the top panel measures the number of genes correctly classified by the indicated fraction of the trained ANNs (*x*-axis, bin range specified in the lower right corner of corresponding confusion array cells). This Expectation Maximization clustering was performed at the K value of 5, previously determined to be optimal for this dataset [9]. Each bin is then broken up across the 5 EM MoDG clusters using a confusion array.
(42 KB PDF)

Figure S3 aobANNs Trained Using Top-Ranked Regulators aobANNs were trained using top-ranked regulators beginning with the top three and continuing through the top 30. Training of these new aobANNs was as described in Methods. Performance of the resulting aobANNs is plotted as a function of the number of top regulators included.
(16 KB PDF)

Figure S4 Network Ranks across Varying Cluster Number (K Values) Results from training ANNs are shown for different clusterings obtained using cluster number (K) over the range from k = 4 to k = 8 clusters. Within each colored heatmap, an individual cell represents a regulator; the position of the cell along the *x*-axis of the plot s specified by K; and the color of the cell indicates the regulator's rank in the original k = 5 network (as shown in Figure 3). Thus the color pattern changes seen reflect the effect and magnitude of change due to use of each different clustering.
(A) An overview of all regulators that shows the overall rank stability of the regulators across variant ANN networks generated.
(B) A higher resolution view of the top-ranked regulators for each variant network. Only the top 50 regulators are shown, and the color spectrum is now adjusted to only span 1–50. Any regulator that was ranked within the top 50 regulators in a mutant network, but was not in the top 50 in the parental K = 5 network, is displayed as white.
(45 KB PDF)

### Accession Numbers

Table of top regulators. Gene descriptions for the top ten positively and negatively associated regulators for each cluster as determined by the ANN weights matrix in Figure 4 with annotations from http://www.yeastgenome.org.

## Abbreviations

ANN: artificial neural network
aob: average-of-bests
SOS: sum-of-squares
