# Supplementary material for: Connectivity in the Yeast Cell Cycle Transcription Network: Inferences from Neural Networks
Source: PLoS Comput Biol. 2006 Dec 22;2(12):e169. doi: 10.1371/journal.pcbi.0020169 (PMC1761652; doi:10.1371/journal.pcbi.0020169)
Supplement: Supplementary file 1 [file pcbi.0020169.sg001.pdf]

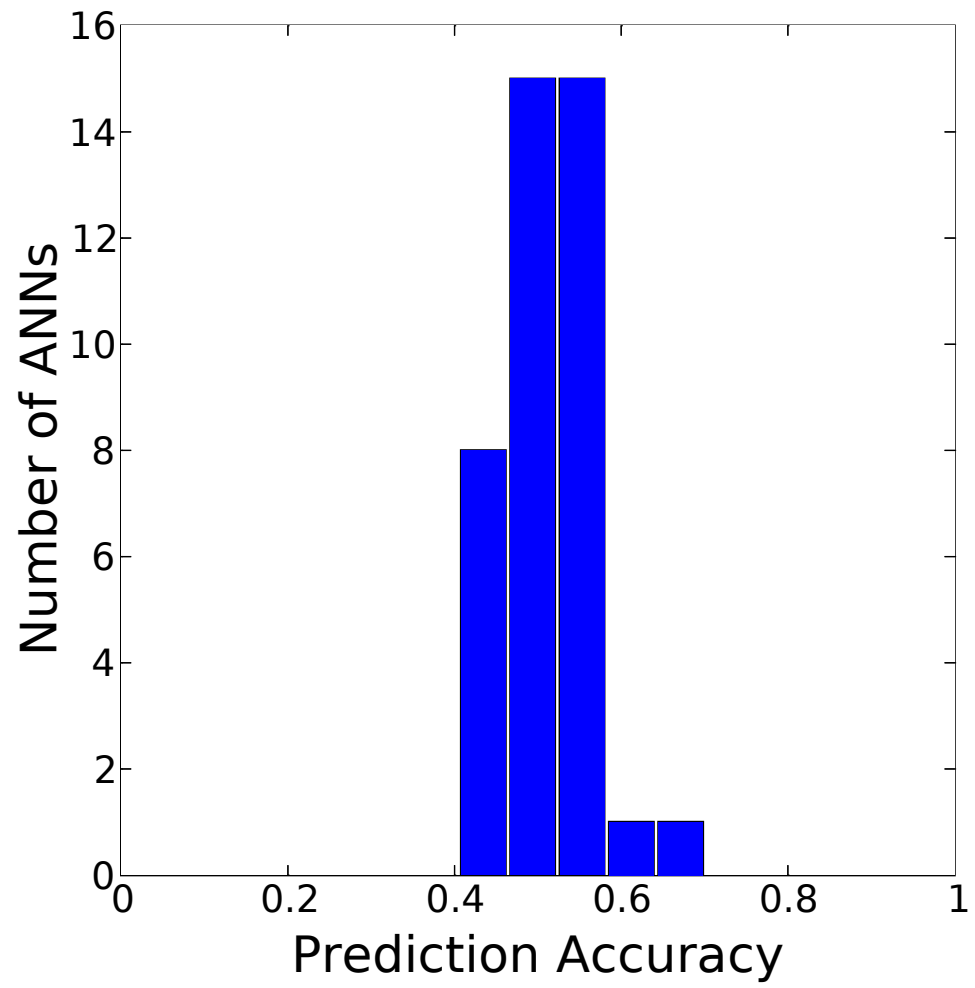

(a) ANN Accuracy

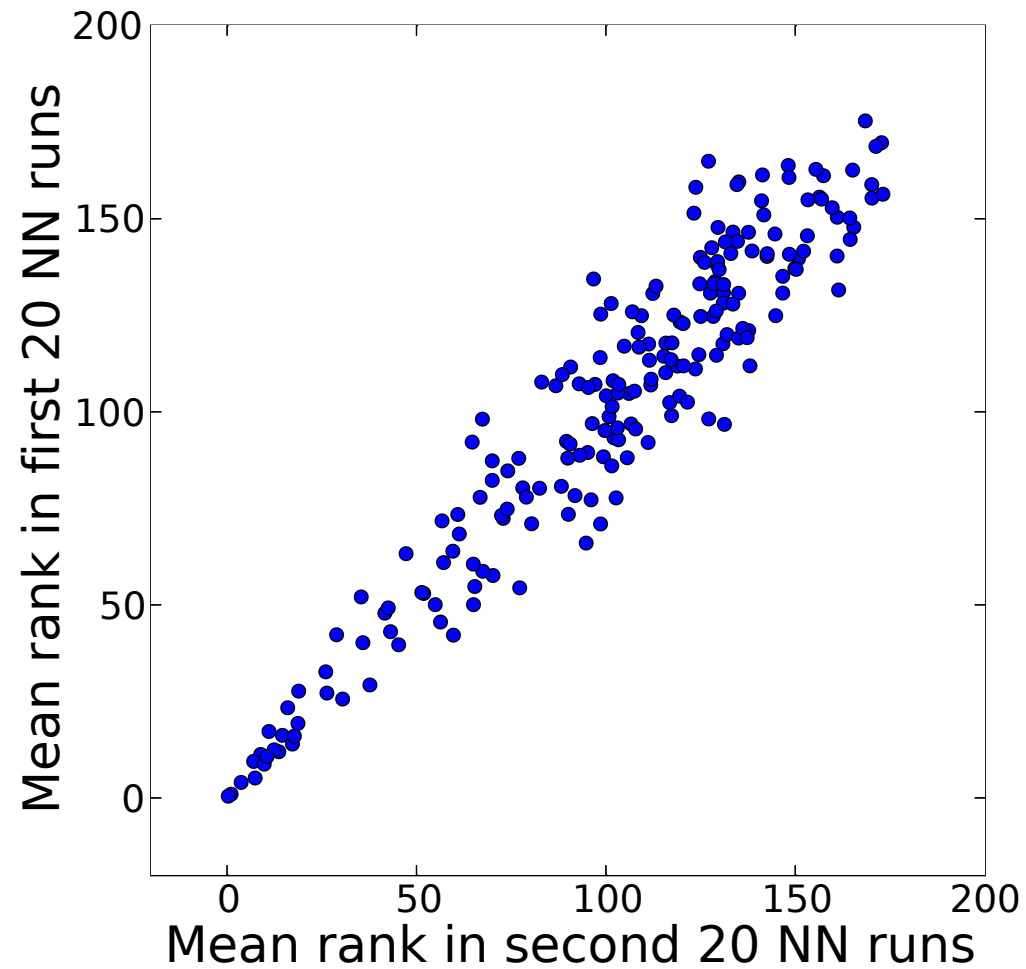

(b) ANN Reproducibility

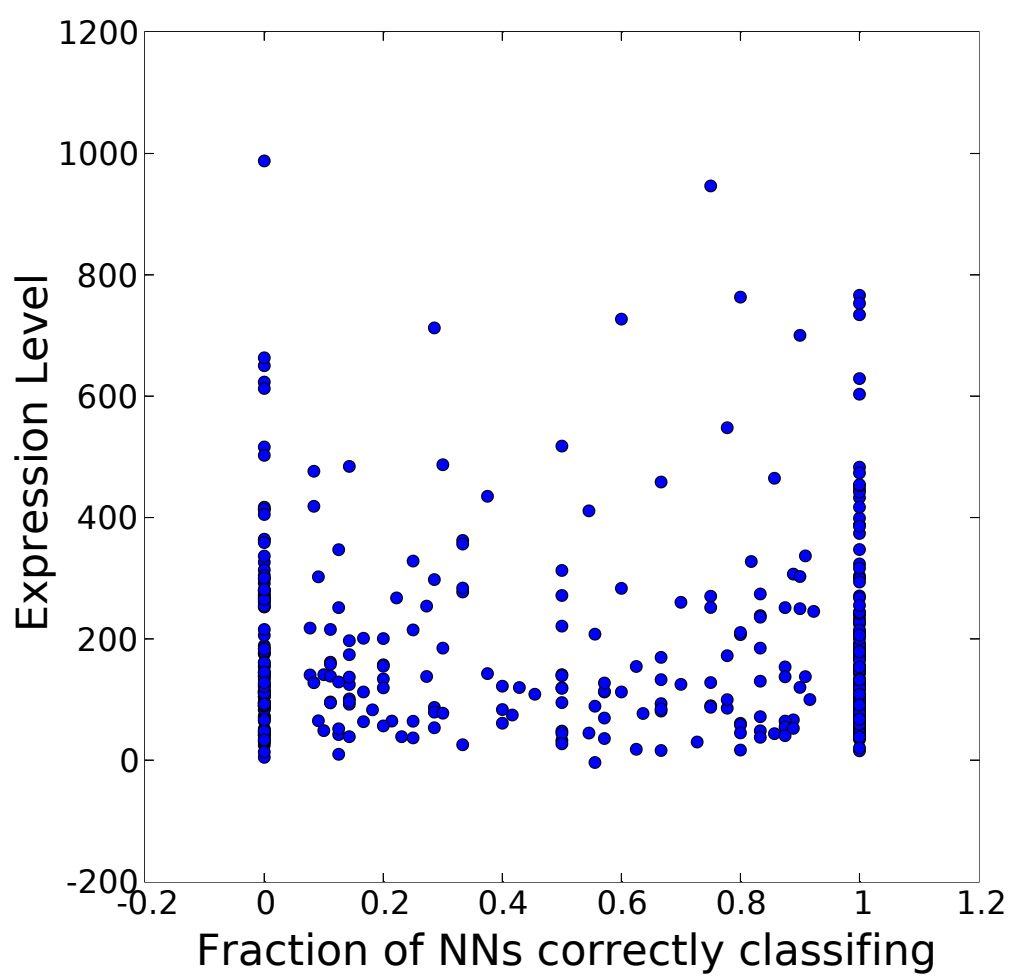

(c) Predictability Vs Expression Level

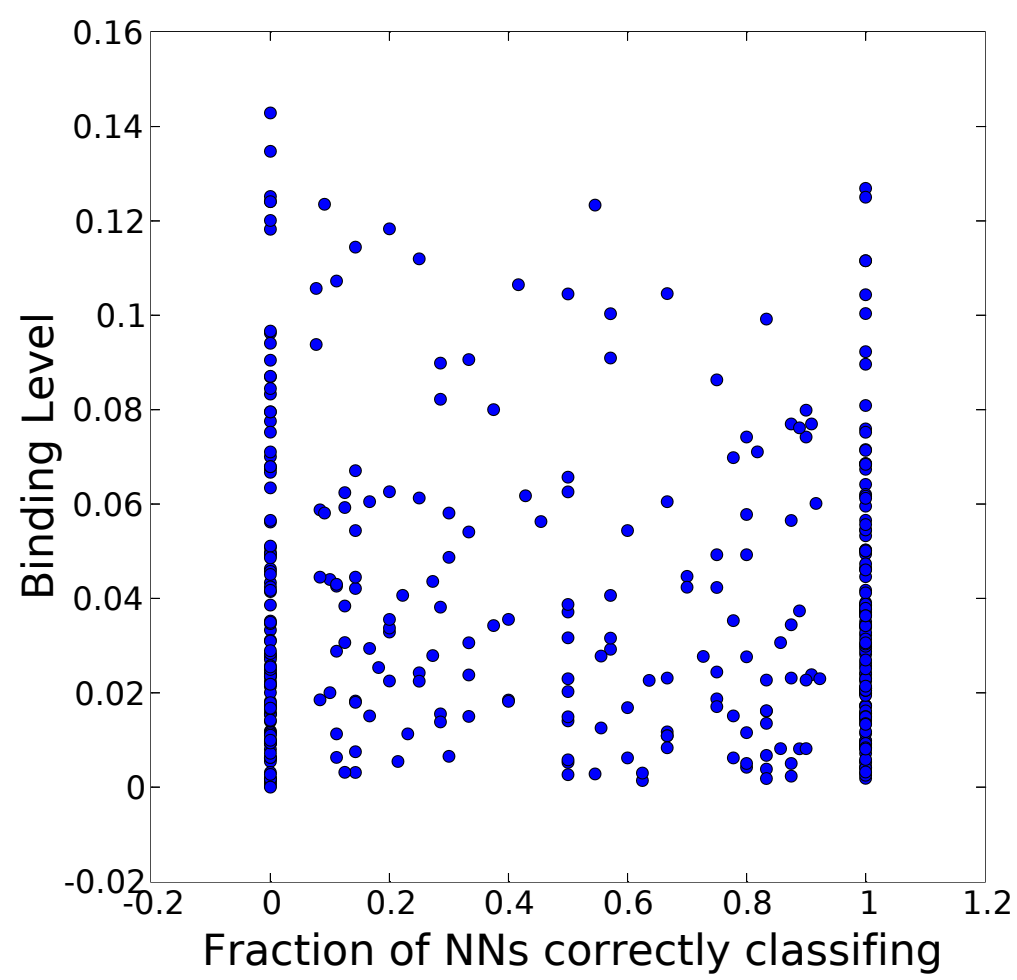

(d) Predictability Vs Binding Level
