# Supplementary material for: Connectivity in the Yeast Cell Cycle Transcription Network: Inferences from Neural Networks
Source: PLoS Comput Biol. 2006 Dec 22;2(12):e169. doi: 10.1371/journal.pcbi.0020169 (PMC1761652; doi:10.1371/journal.pcbi.0020169)
Supplement: Supplementary file 2 [file pcbi.0020169.sg002.pdf]

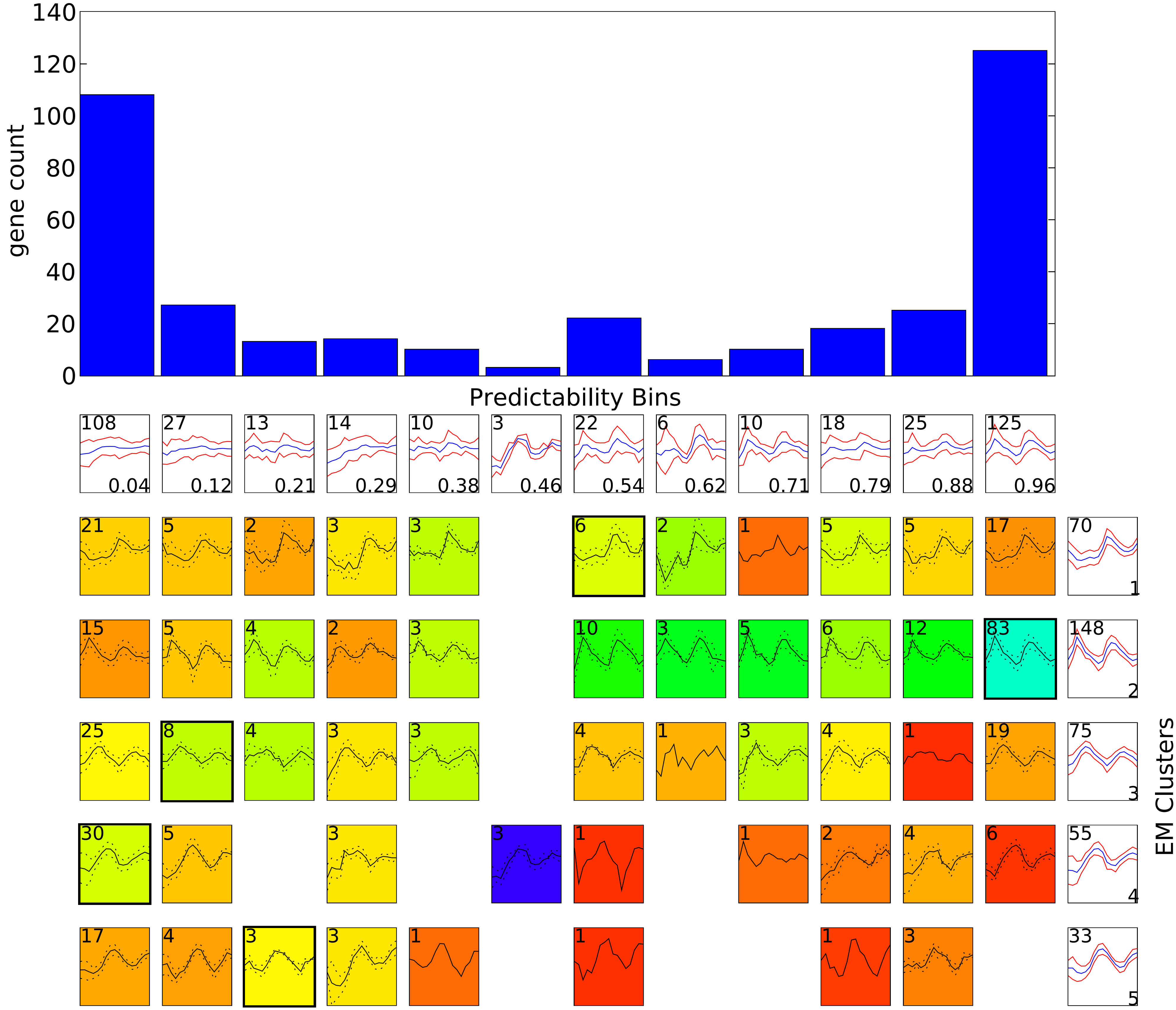
gene count
Predictability Bins
EM Clusters
0.04
0.12
0.21
0.29
0.38
0.46
0.54
0.62
0.71
0.79
0.88
0.96
