# Supplementary material for: Connectivity in the Yeast Cell Cycle Transcription Network: Inferences from Neural Networks
Source: PLoS Comput Biol. 2006 Dec 22;2(12):e169. doi: 10.1371/journal.pcbi.0020169 (PMC1761652; doi:10.1371/journal.pcbi.0020169)
Supplement: Supplementary file 3 [file pcbi.0020169.sg003.pdf]

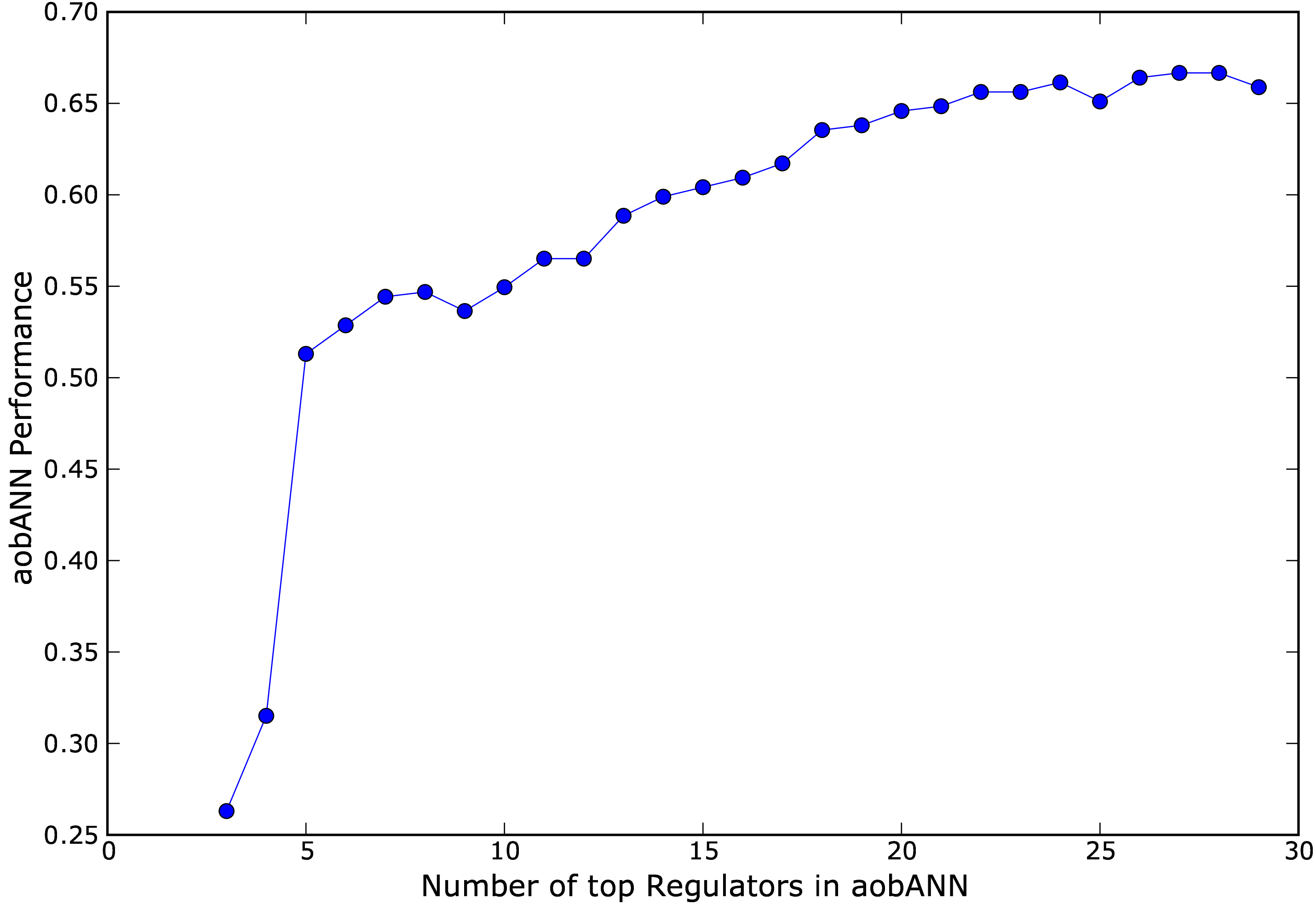

0.70
0.65
0.60
0.55
0.50
0.45
0.40
0.35
0.30
0.25
aobANN Performance
0
5
10
15
20
25
30
Number of top Regulators in aobANN
