# Supplementary material for: Connectivity in the Yeast Cell Cycle Transcription Network: Inferences from Neural Networks
Source: PLoS Comput Biol. 2006 Dec 22;2(12):e169. doi: 10.1371/journal.pcbi.0020169 (PMC1761652; doi:10.1371/journal.pcbi.0020169)
Supplement: Supplementary file 4 [file pcbi.0020169.sg004.pdf]

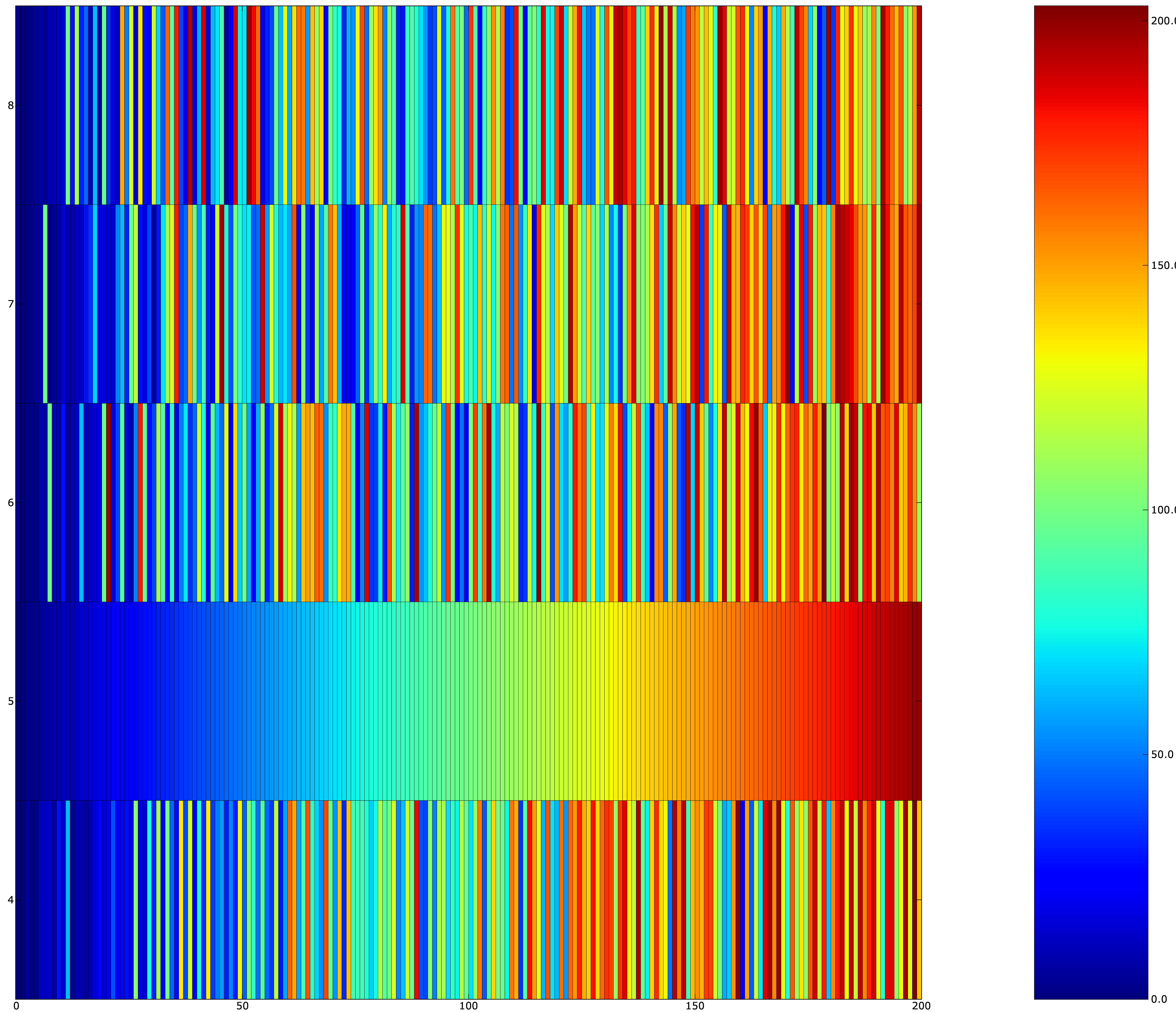

8
7
6
5
4
0
50
100
150
200
200.0
150.0
100.0
50.0
0.0

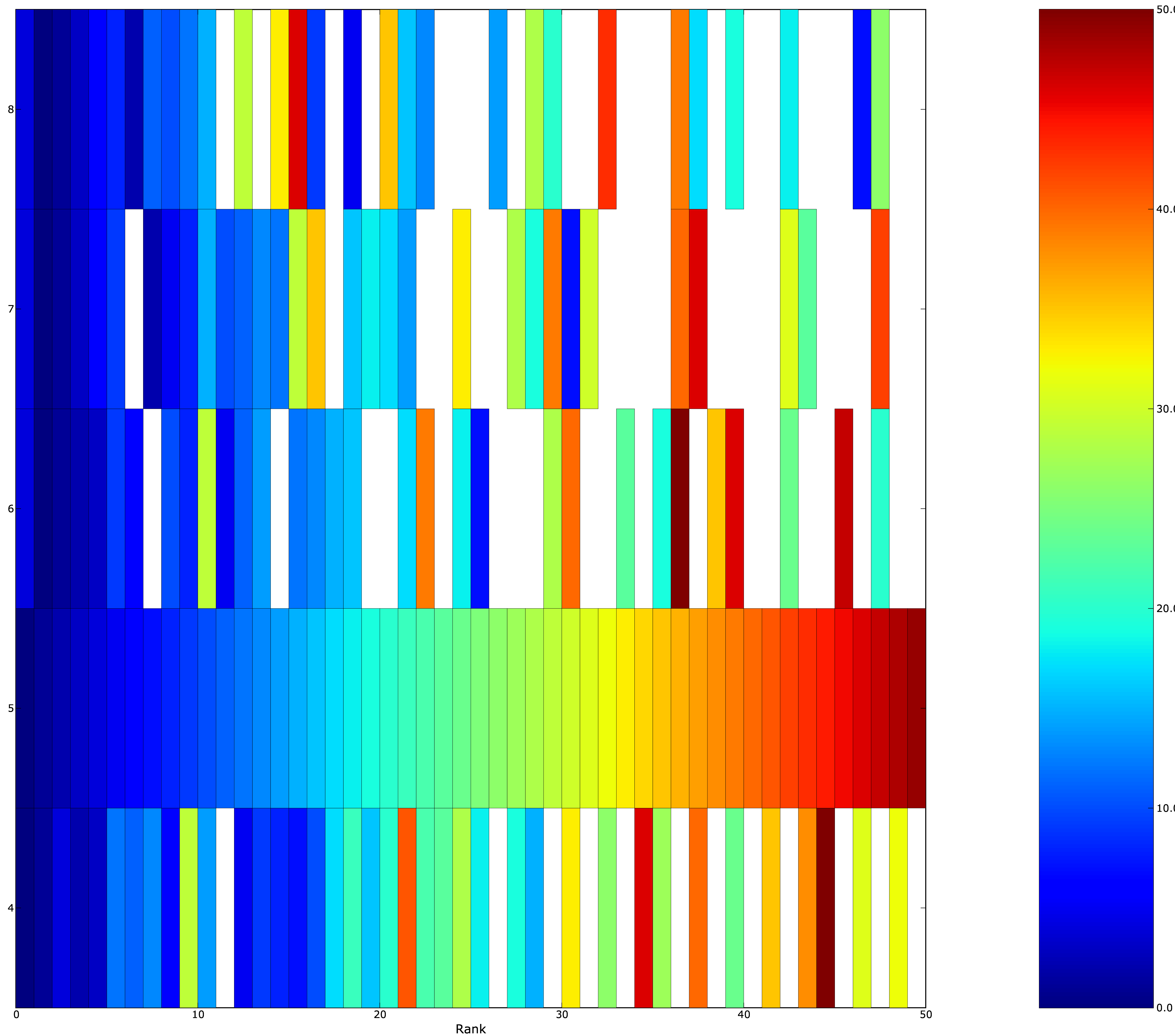

8
7
6
5
4
0
10
20
30
40
50
Rank
50.0
40.0
30.0
20.0
10.0
0.0
